# No Detectable Maternal Effects of Elevated CO_2_ on *Arabidopsis thaliana* Over 15 Generations

**DOI:** 10.1371/journal.pone.0006035

**Published:** 2009-06-25

**Authors:** Nianjun Teng, Biao Jin, Qinli Wang, Huaiqing Hao, Reinhart Ceulemans, Tingyun Kuang, Jinxing Lin

**Affiliations:** 1 Key Laboratory of Photosynthesis and Environmental Molecular Physiology, Institute of Botany, Chinese Academy of Sciences, Beijing, China; 2 College of Horticulture, Nanjing Agricultural University, Nanjing, China; 3 College of Horticulture and Plant Protection, Yangzhou University, Yangzhou, China; 4 Department of Biology, University of Antwerpen, Antwerp, Belgium; University of Sheffield, United Kingdom

## Abstract

Maternal environment has been demonstrated to produce considerable impact on offspring growth. However, few studies have been carried out to investigate multi-generational maternal effects of elevated CO_2_ on plant growth and development. Here we present the first report on the responses of plant reproductive, photosynthetic, and cellular characteristics to elevated CO_2_ over 15 generations using *Arabidopsis thaliana* as a model system. We found that within an individual generation, elevated CO_2_ significantly advanced plant flowering, increased photosynthetic rate, increased the size and number of starch grains per chloroplast, reduced stomatal density, stomatal conductance, and transpiration rate, and resulted in a higher reproductive mass. Elevated CO_2_ did not significantly influence silique length and number of seeds per silique. Across 15 generations grown at elevated CO_2_ concentrations, however, there were no significant differences in these traits. In addition, a reciprocal sowing experiment demonstrated that elevated CO_2_ did not produce detectable maternal effects on the offspring after fifteen generations. Taken together, these results suggested that the maternal effects of elevated CO_2_ failed to extend to the offspring due to the potential lack of genetic variation for CO_2_ responsiveness, and future plants may not evolve specific adaptations to elevated CO_2_ concentrations.

## Introduction

Over the next century, the atmospheric CO_2_ concentration is projected to rise from the current level of about 370 parts per million (ppm) to between 540 and 970 ppm [1]. Given that CO_2_ is the raw material of photosynthesis, this global change will have profound effects on the structure and function of future plant populations [2]–[8]. A typical experimental approach used in most CO_2_ experiments to predict how future plants will respond to these changes is to expose individual plants or plant communities to ambient and elevated CO_2_ within a part, or one generation and compare their responses [3], [9]–[15]. Even several long-term studies of trees, lasting up to 30 years, are still limited to one generation [16]–[18]. A major assumption in such experiments is that the responses of plants to elevated CO_2_ within one generation can be similar to those observed over many generations. However, an experimental test of the assumption is currently unavailable.

Earlier investigations frequently used seed-propagated annuals as the experimental materials and revealed that maternal environmental conditions became manifest in seed characters which, in turn, may influence the performance of the offspring by altering seed germination, seedling survival and growth [19]–[24]. In other words, plastic response to the environment may extend to an individual's offspring, influencing offspring trait expression [20], [25]. As a consequence, most of the previous studies investigating the responsiveness of plants to elevated CO_2_ within one generation failed to notice the maternal effects on the offspring growth and the predictions based on the results from such experiments can be challenged. It is necessary, therefore, to illustrate the importance and necessity of examining CO_2_ response of plants over more than one generation to make accurate predictions about biological consequences of increasingly rising atmospheric CO_2_ concentration.

Here, we carried out a fifteen-generation selection experiment and a reciprocal sowing experiment (over 5 years in total) using *Arabidopsis thaliana* (wild-type Columbia) as a model plant to examine multi-generation maternal effects of elevated CO_2_ on plant growth and development. *Arabidopsis* is an ideal plant for investigating this issue for three main reasons. First, the short generation time allows us to study the responses of many generations over a reasonably short period of time. Second, its small size makes it possible to grow a large population of plants under controlled CO_2_ conditions. Finally, the life history and allocation strategy of *Arabidopsis* is common to numerous annuals that have a short generation time and allocate a high proportion of their resources to reproduction. Thus, the responses of *Arabidopsis* may provide valuable insights into the responses of various annuals to the rising atmospheric CO_2_ concentration [2], [26].

On the basis of our studies over the past several years [27]–[29], this study is part of a series examining multi-generational maternal effects of elevated CO_2_ on *Arabidopsis*. Our overall aim is to reveal the physiological, cytological, and reproductive responses to elevated CO_2_, to determine if elevated CO_2_ can produce maternal effects on plant growth and development across fifteen generations, and to test if the responses of plants to elevated CO_2_ within one generation will be similar to those observed over many generations.

## Results

### Reproductive responses to elevated CO_2_

**Figure 1:**
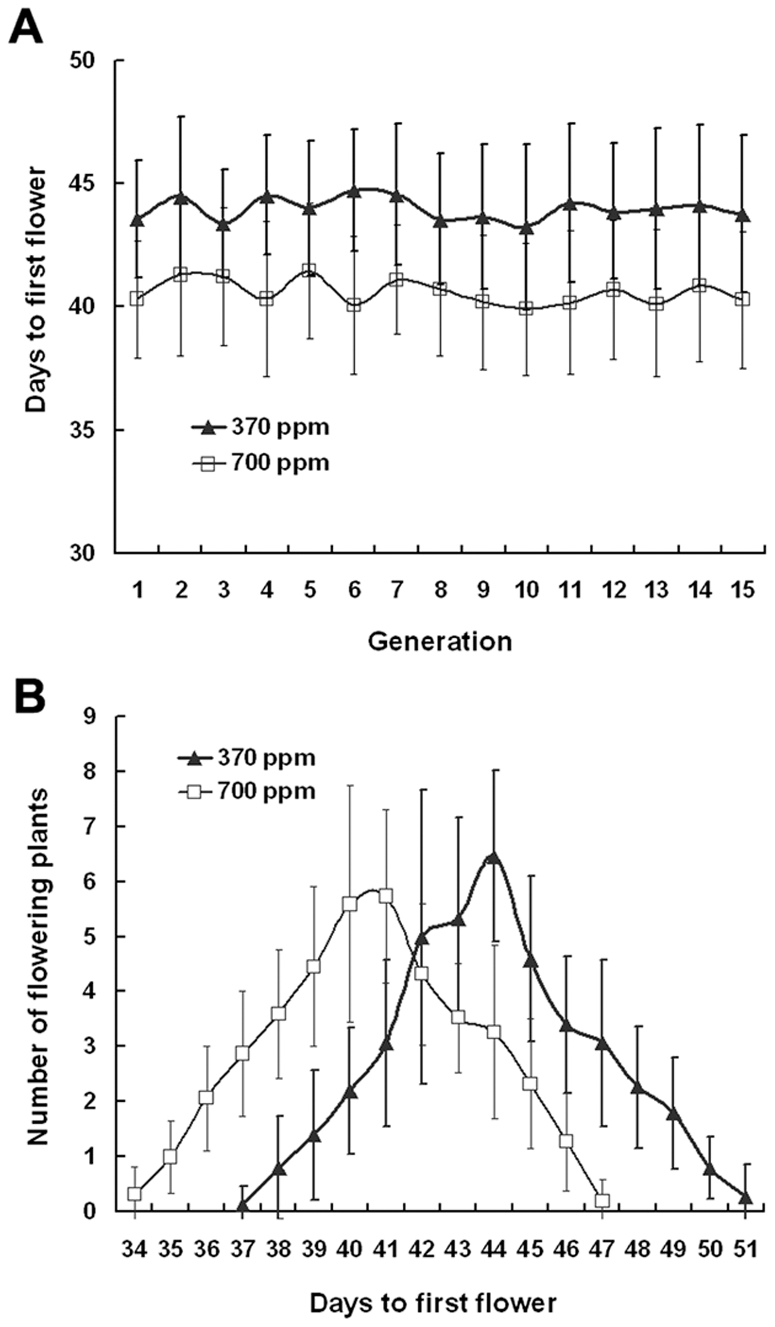
Effects of elevated CO_2_ on the flowering time of Arabidopsis thaliana over 15 generations. A, On average, plants grown in elevated CO_2_ flowered significantly earlier than those grown in ambient CO_2_ concentrations within each generation. B, The number of flowering plants per day was recorded in ambient and elevated CO_2_ across 15 generations. Error bars represent the standard deviation of the mean.

The date of opening of the first flower was significantly affected by the CO_2_ treatment (Figure 1). On average, plants grown at elevated CO_2_ concentrations flowered about three days earlier than those grown at ambient CO_2_ concentrations in each generation. However, within the same CO_2_ treatment, the average number of days to first flowering in any two generations was similar, and no significant difference was observed in flowering time among the 15 generations. For example, the average number of days to first flowering in any generation averaged around 40.5 for the populations at elevated CO_2_ and 44 for those at ambient CO_2_. Taken together, within an individual generation, CO_2_ treatment resulted in a significant change in flowering time, whereas no significant changes in flowering time were detected among generations within the same CO_2_ treatment.

**Figure 2:**
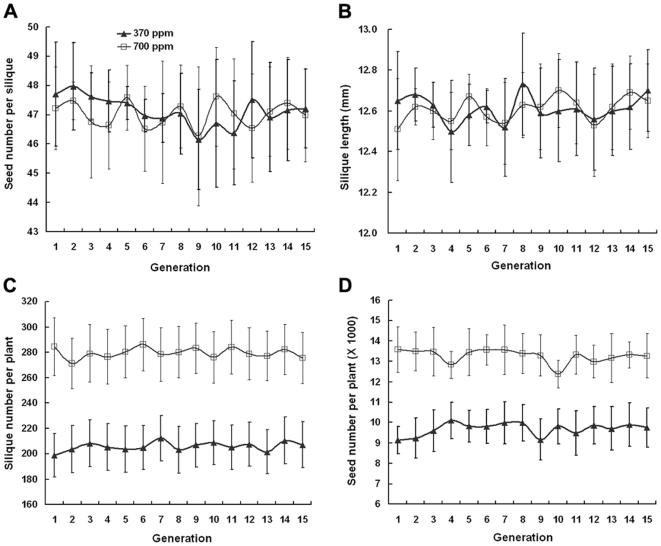
Effects of elevated CO_2_ on silique length and number of siliques and seeds. A and B, elevated CO_2_ had no significant effect on the number of seeds per silique or silique length. C and D, elevated CO_2_ significantly increased the number of siliques and the number of seeds per plant. Error bars represent the standard deviation of the mean.

The number of seeds per silique and silique length did not change significantly in response to CO_2_ treatment (Figure 2A and B). However, we detected significant treatment effects on the number of siliques and the number of seeds per plant (Figure 2C and D). The average number of siliques and seeds per plant across generations in the elevated treatment were significantly higher than those in the ambient treatment. For example, the average number of siliques and seeds per plant exposed to elevated CO_2_ concentrations were about 36% and 37% higher, respectively, than those exposed to ambient CO_2_ concentrations. In the same CO_2_ treatment, however, the number of siliques and the number of seeds per plant did not differ significantly across generations. Across generations, the average number of siliques per plant in the elevated and ambient CO_2_ treatments averaged around 280 and 206, respectively. Similarly, the average number of seeds per treatment across the 15 generations averaged around 13,000 for elevated CO_2_ and 9,500 for ambient CO_2_.

**Figure 3:**
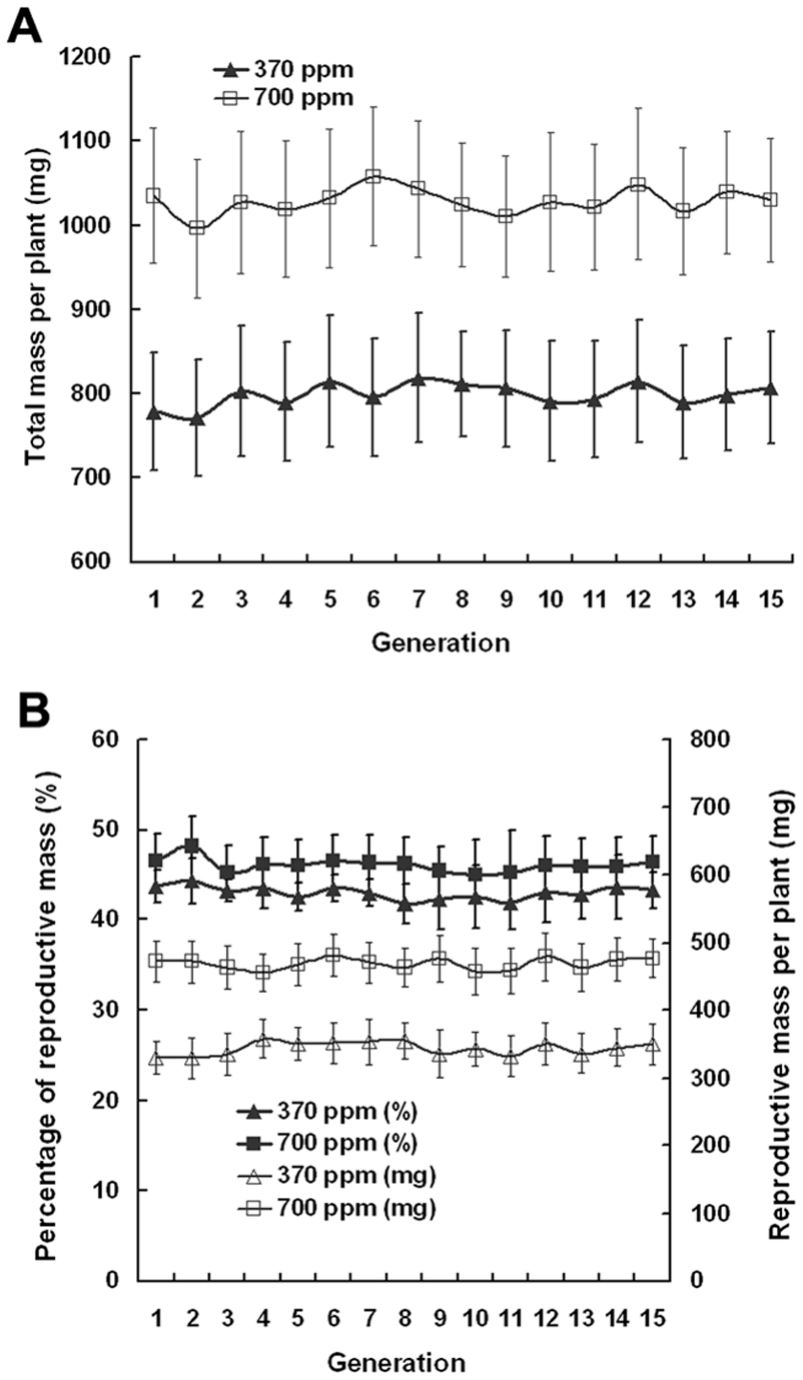
Effects of elevated CO_2_ on total and reproductive mass. Elevated CO_2_ significantly increased total mass (A), reproductive mass, and the relative proportion (%) of reproductive mass per plant (B). Error bars represent the standard deviation of the mean.

The reproductive mass, total mass per plant, and percentage of reproductive mass per plant were higher with elevated CO_2_ than with ambient CO_2_ concentrations. However, these traits did not change significantly across generations in either treatment (Figure 3). On average, the total mass and reproductive mass per plant when grown at elevated CO_2_ levels were about 1020 and 470 mg, respectively, representing increases of about 27% and 36% over those grown at ambient CO_2_ concentrations. A similar trend was observed for the relative proportion (%) of reproductive mass per plant, increasing from about 43% in ambient CO_2_ to about 46% in elevated CO_2_, indicating that more mass was allocated to reproductive growth at elevated CO_2_ concentrations. Within the same CO_2_ treatment, each of these traits was similar across 15 generations, demonstrating that changes in the traits induced by elevated CO_2_ failed to transfer from one generation to the next via reproduction.

### Responses of stomatal and photosynthetic traits to elevated CO_2_

Elevated CO_2_ significantly reduced stomatal density on both adaxial and abaxial leaf surfaces of plants grown in generations 1, 8, and 15 and in reciprocal sowing experiments (Table 1). For example, on average, stomatal density on the adaxial and abaxial leaf surfaces of plants in these generations was significantly decreased by 15.5% and 12.1% with elevated CO_2_, respectively. However, stomatal density did not change significantly among these generations in either treatment. For instance, stomatal density on the adaxial leaf surface averaged about 214 per mm^2^ at ambient CO_2_ concentrations, ranging from 207.2 to 219.4, and approximately 181 at elevated CO_2_ concentrations, ranging from 175.4 to 188.4.

**Table 1:** Stomatal density of leaves of *Arabidopsi*s plants grown at elevated or ambient CO_2_ in different generations.

Stomatal density		Adaxial surface		decrease	Abaxial surface		decrease
Treatment		AC	EC		AC	EC	
Generation	1	216.8±12.6a	176.3±10.0b	18.7%	236.3±12.8a	204.3±10.5b	13.5%
	8	207.2±12.3a	183.2±9.7b	11.6%	231.7±12.6 a	202.8±11.5b	12.5%
	15	219.4±11.6a	188.4±10.3b	14.1%	237.1±11.8 a	210.7±10.8b	11.1%
	SA*	211.6±11.4a	180.5±9.2b	14.7%	240.2±12.1a	213.2±11.8b	11.2%
	SE*	215.5±11.1a	175.4±10.1b	18.6%	234.7±10.9a	205.9±11.2b	12.3%
P-value		0.145–0.869	0.078–0.886	/	0.307–0.921	0.197–0.838	/
Average		214.1	180.8	15.5%	236	207.4	12.1%

The values given indicate means±SD from five plants. Three fully expanded rosette leaves at stage 5.0 were sampled from each of five plants and twenty separate fields were analyzed in each leaf. Mean values were compared by t-test.

* The seeds used in the reciprocal sowing experiments were from the fifteenth generation grown in ambient CO_2_ (SA) and elevated CO_2_ (SE).

Abbreviations: AC: Ambient CO_2_; EC: Elevated CO_2_.

Elevated CO_2_ also significantly reduced stomatal conductance and transpiration rate, but increased the photosynthetic rate of *Arabidopsis* leaves in generations 1, 8, and 15 and in the reciprocal sowing experiments (Table 2). Relative to that in ambient CO_2_, stomatal conductance and transpiration rate in elevated CO_2_ were on average reduced by about 41.9% and 34.1%, respectively. However, compared to ambient CO_2_, elevated CO_2_ significantly increased photosynthetic rate with an average of 17.1% in these generations. Although elevated CO_2_ significantly affected stomatal conductance, transpiration rate, and photosynthetic rate within each generation, the three traits did not change significantly among these generations in either treatment. For example, stomatal conductance at elevated CO_2_ ranged from 220.4 to 239.4 mmol m^−2^ s^−1^ in these generations, with an average of about 227.9, and no significant difference was detected in stomatal conductance among these generations.

**Table 2:** Stomatal conductance, transpiration rate and photosynthetic rate of leaves of *Arabidopsis* plants grown at elevated or ambient CO_2_ in different generations.

Photosynthetic features		Stomatal conductance (mmol m^−2^ s^−1^)		decrease	Transpiration rate (mmol m^−2^ s^−1^)		decrease	Photosynthetic rate (µmol m^−2^ s^−1^)		increase
Treatment		AC	EC		AC	EC		AC	EC	
Generation	1	385.6±23.2a	220.4±14.0b	42.8%	8.16±0.41a	5.18±0.24b	36.5%	14.1±1.2a	16.3±1.4b	15.6%
	8	410.4±25.5a	239.4±15.6b	41.7%	8.32±0.40a	5.46±0.25b	34.4%	14.8±1.5a	17.2±1.3b	16.2%
	15	377.8±21.6a	221.6±13.9b	41.3%	7.79±0.39a	5.15±0.21b	33.9%	13.5±1.4a	15.8±1.5b	17.0%
	SA*	382.0±20.4a	227.8±13.3b	40.4%	7.91±0.45a	5.34±0.28b	32.5%	13.9±1.4a	16.6±1.3	19.4%
	SE*	406.2±22.5a	230.4±16.1b	43.3%	8.05±0.36a	5.38±0.31b	33.2%	14.4±1.6a	16.9±1.7b	17.4%
P-value		0.061–0.801	0.078–0.895	/	0.067–0.650	0.067–0.838	/	0.204–0.813	0.162–0.808	/
Average		392.4	227.9	41.9%	8.05	5.30	34.1%	14.1	16.6	17.1%

The values given indicate means±SD from five plants. Three fully expanded rosette leaves at stage 5.0 were sampled from each of five plants were analyzed for stomatal conductance, transpiration rate and photosynthetic rate. Mean values were compared by *t*-test.

* The seeds used in the reciprocal sowing experiments were from the fifteenth generation grown in ambient CO_2_ (SA) and elevated CO_2_ (SE).

Abbreviations: AC: Ambient CO_2_; EC: Elevated CO_2_.

### Responses of leaf ultrastructure to elevated CO_2_

**Figure 4:**
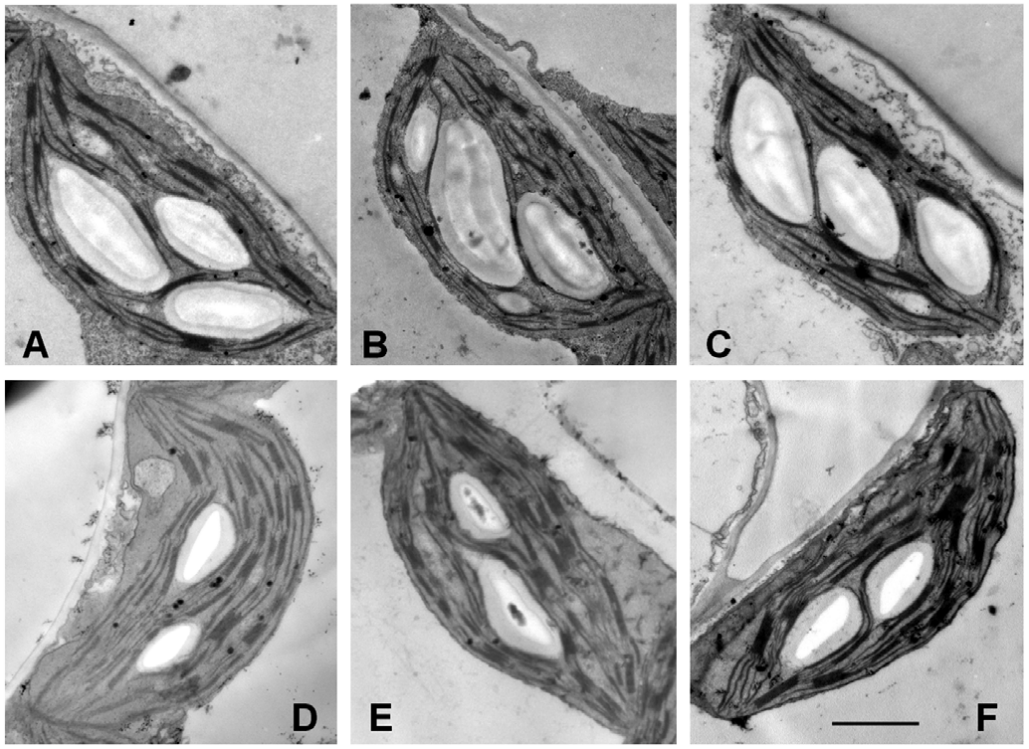
Effects of elevated CO_2_ on leaf chloroplast ultrastructure during different generations. Plants were grown in elevated CO_2_ in generations 1 (A), 8 (B), and 15 (C), and under ambient CO_2_ in generations 1 (D), 8 (E), and 15 (F). Note that more and larger starch grains were observed in chloroplasts of elevated-CO_2_ grown leaves than in chloroplasts of ambient-CO_2_ grown leaves in any of the three generations. However, there was no significant difference in the number and size of starch grains in either treatment among the three generations. Scale bar = 1 µm.

Relative to ambient CO_2_, elevated CO_2_ concentrations on average significantly increased the number of starch grains per chloroplast profile and area per starch grain by 42.4% and 51.9%, respectively, in leaves of plants grown in generations 1, 8, and 15 and in reciprocal sowing experiments (Table 3 and Figure 4). However, each of the traits did not change significantly among these generations when exposed to either ambient CO_2_ or elevated CO_2_ (Table 3 and Figure 4). For example, the number of starch grains per chloroplast profile averaged around 1.95 at ambient CO_2_ concentrations, ranging from 1.87 to 2.05, and around 2.77 with elevated CO_2_, ranging from 2.68 to 2.87. The change in area per starch grain also followed a similar pattern.

**Table 3:** Chloroplast feature of leaves of *Arabidopsis* plants grown at elevated or ambient CO_2_ in different generations.

Chloroplast feature		Number of starch grains per chloroplast profile		increase	Area per starch grain (µm^2^)		increase
Treatment		AC	EC		AC	EC	
Generation	1	1.94±1.28a	2.76±1.41b	42.3%	0.91±0.49a	1.39±0.73b	52.7%
	8	1.90±1.34a	2.73±1.38b	43.5%	0.86±0.52a	1.24±0.66b	44.2%
	15	1.99±1.41a	2.87±1.43b	44.3%	0.81±0.57a	1.29±0.72b	59.3%
	SA*	2.05±1.29a	2.83±1.38b	37.9%	0.93±0.53a	1.36±0.69	46.2%
	SE*	1.87±1.22a	2.68±1.31b	43.8%	0.84±0.51a	1.32±0.61b	57.1%
P-value		0.074–0.750	0.103–0.792	/	0.079–0.761	0.083–0.723	/
Average		1.95	2.77	42.4%	0.87	1.32	51.9%

The values given indicate means±SD from five plants. Number of starch grains per chloroplast profile was determined according to 300 chloroplasts. Area per starch grain was determined from 150 starch grains. The fully expanded rosette leaves were sampled at stage 5.0. Mean values were compared by *t*-test.

* The seeds used in the reciprocal sowing experiments were from the fifteenth generation grown in ambient CO_2_ (SA) and elevated CO_2_ (SE).

Abbreviations: AC: Ambient CO_2_; EC: Elevated CO_2_.

### Evidence from reciprocal sowing experiments

**Figure 5:**
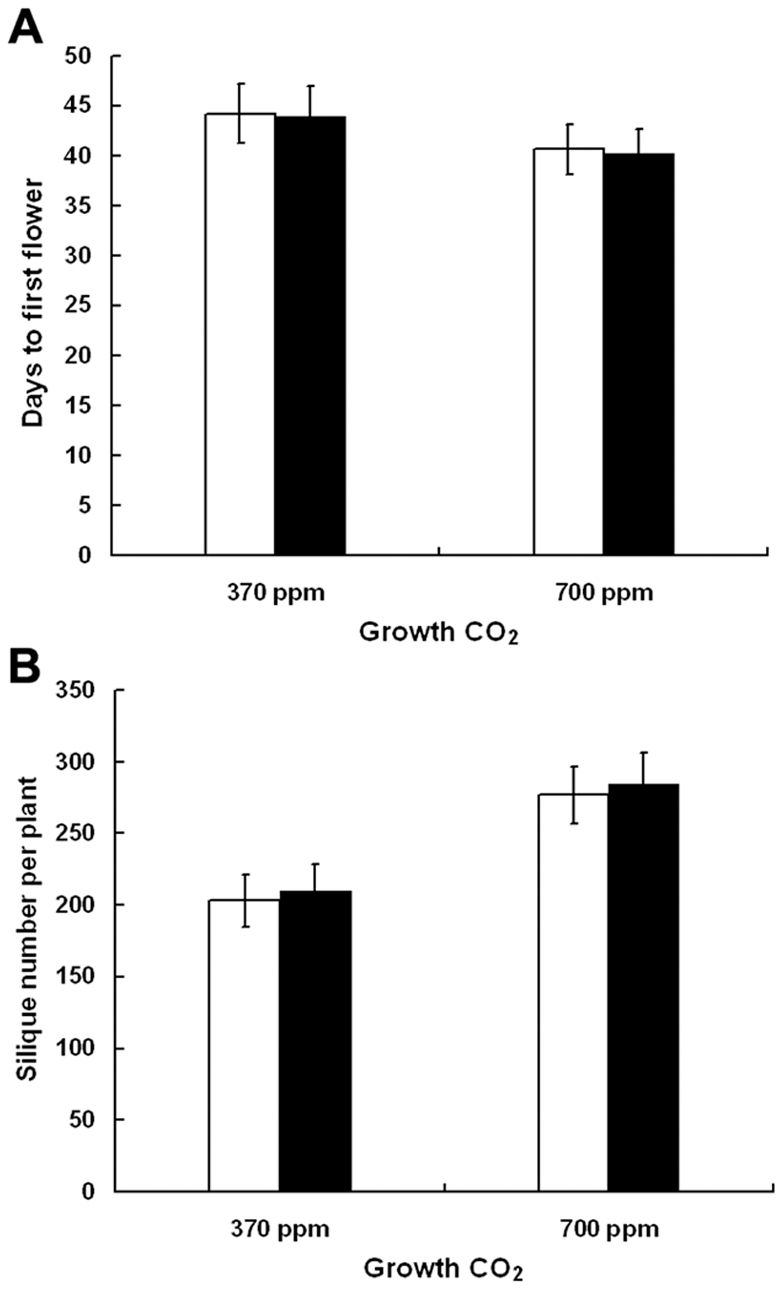
Days to first flower and number of siliques during the reciprocal sowing experiments. Open and solid bars indicate that seeds were obtained from the plants of the fifteenth generation grown at ambient and elevated CO_2_ concentrations, respectively. Seed source had no significant effect on days to first flower of plants (A) or number of siliques per plant (B).

To evaluate whether *Arabidopsis* plants exhibited an adaptive response to elevated CO_2_, we conducted a reciprocal sowing experiment in which seeds from the fifteenth generation in each treatment were grown at both ambient and elevated CO_2_ concentrations. As a result, we did not detect significant interactions between the maternal CO_2_ environment and the CO_2_ transplant environment (Figure 5, and Tables 1, 2, 3). Plants from fifteenth-generation seeds grown under ambient and elevated CO_2_ were similar, with no significant differences in several traits between the two populations under either CO_2_ treatment regime. In other words, at a given CO_2_ concentration, the traits of both populations were similar to those observed at that CO_2_ level during the selection experiment. For example, the average time to first flowering was about 44 days in both populations when grown at ambient CO_2_ during the sowing experiment and was similar to that at ambient CO_2_ during the selection experiment (Figures 1A, 5A). Similarly, the average time to first flowering was about 40.5 days in both populations when plants were grown at elevated CO_2_, which was not significantly different from that at elevated CO_2_ during the selection experiment (Figures 1A, 5A). There were similar patterns for the change in silique number per plant, stomatal density in both adaxial and abaxial leaf surfaces, stomatal conductance, transpiration rate, photosynthetic rate, and chloroplast features during the sowing experiment (Figure 5, and Tables 1, 2, 3).

## Discussion

Many studies have investigated plant responses to elevated CO_2_ on the ecosystem, community, population, plant, leaf, physiological, biochemical, and molecular levels over the past two decades, most of which were carried out on plants grown only for a single generation of plants [2], [24], [29]–[31]. The main results from those studies indicate that elevated CO_2_ generally accelerates plant growth and development [27], [32], [33], advances flowering time [31], [34], [35], reduces stomatal density, stomatal conductance, and transpiration rate [6], [27], [36], increases photosynthetic rate and carbohydrate content [4], [27], [31], and enhances reproductive output by altering flower number, fruit set, and seed production [13]. For example, Woodward and Kelly [37] reported an average reduction in stomatal density of 14.3% for 100 species grown under CO_2_ enrichment. In addition, Jablonski et al. [13] used meta-analysis to integrate data on eight reproductive characteristics from 159 CO_2_ enrichment papers that provided information on 79 species and found that, on average, elevated CO_2_ increased fruits, seeds, and total seed mass by 19%, 18%, and 25%, respectively. In the present study, we found that elevated CO_2_ significantly advanced the flowering time of *Arabidopsis*, resulted in more siliques and seeds, reduced stomatal density, stomatal conductance, and transpiration rate, and increased photosynthetic rate and the size and number of starch grains in the chloroplast. These results are consistent with previous reports. However, silique length and number of seeds per silique were not influenced by elevated CO_2_, and it is possible that the two traits are less plastic than other traits in response to elevated CO_2_. Given that the number of seeds per silique changed only slightly with CO_2_ enrichment, the significant increase in the number of seeds per plant was mainly attributed to a significant increase in the number of siliques per plant.

In the current study, it was of great interest to find that within each of the 15 generations, elevated CO_2_ had significant effects on many traits including flowering time, total and reproductive mass, stomatal density and conductance, transpiration rate, and photosynthetic rate, but each of these traits in any two of the 15 generations was similarly responsive to elevated CO_2_. In other words, no significant difference was observed in each of these traits across the 15 generations within the same CO_2_ treatment, indicating that maternal CO_2_ had no significant effect on her offspring performance or transgenerational effects of CO_2_ were relatively small in this genotype. Our results led us to reject our hypothesis that plants in generation m+n would be more responsive to elevated CO_2_ than those in generation m (m≥1, n≥1). For instance, according to this hypothesis, if the days to first flowering for elevated CO_2_-grown plants in generation 1 averaged around 40.5, then the days for elevated CO_2_-grown plants in generation 1+n (n≥1) would be significantly shorter than 40.5. Our initial hypothesis was based on the assumption that elevated CO_2_ can exert a selective pressure on plants sufficient to produce genetic variation, and maternal responses to elevated CO_2_ may extend to the offspring and even accumulate via reproduction, influencing the offspring trait expression [2], [25]. This assumption, however, proved to be false, since all 15 generations were nearly equally responsive to elevated CO_2_ or maternal CO_2_ did not produce significant effects on the offspring. The contradictory resulted largely from the fact that elevated CO_2_ may generate immediate phenotypic change via phenotypic plasticity, but fails to produce genetic change [9], [23], [38]. Therefore, our results from 15 generations demonstrated that elevated CO_2_ significantly affected many traits and enhanced fitness of *Arabidopsis* plants within a single generation, but maternal effects of elevated CO_2_ did not influence the offspring trait expression largely due to the potential lack of genetic variation for CO_2_ responsiveness. Moreover, the results from the reciprocal sowing experiments confirmed that elevated CO_2_ did not produce detectable maternal effects on *Arabidopsis* even after 15 generations

Several studies have used a variety of plant species, including *Arabidopsis thaliana* [2], *Sanguisorba minor* [19], *Bromus erectus* [23], *Cerastium glomeratum*, *Leontodon saxatilis*, *Poa pratensis* and *Trifolium repens* [39], to investigate maternal effects of elevated CO_2_ on plant growth, most of which focused on the responses within a single generation. For example, Steinger et al. [23] reported the maternal and direct effects of elevated CO_2_ on seed provisioning, germination and seedling growth in *B. erectus* and found that seed germination rate and seedling size were not significantly affected by elevated maternal CO_2_. Similar results were also observed in algae, *C. glomeratum* and *P. pratensis* [39]–[41]. Our results from 15 generations and the reciprocal sowing experiments demonstrated that elevated CO_2_ failed to produce detectable maternal effects on th*e Arabidopsis* plants. Although elevated CO_2_ cannot produce significant maternal effects on the offspring or transgenerational effects of elevated CO_2_ are very small, the mechanism for the non-detectable maternal effects is poorly understood. A possible explanation for this is that the advantages obtained such as increased seed mass at elevated maternal CO_2_ may be offset by the reduced concentration of nitrogen (and possibly other nutrients) or the increase in the C∶N ratio [23]. Another explanation may be that the selective pressure of elevated CO_2_ concentration is not high enough to generate genetic changes, unlike certain other factors including heavy metal contamination, drought, biological invasion, and global warming [38], [42]–[44].

In summary, elevated CO_2_ had a significant positive impact on some reproductive, photosynthetic, and cellular traits of *Arabidopsis* in the first generation, but the effect was not significantly strengthened after additional generations at elevated CO_2_. In addition, those traits measured at elevated CO_2_ were restored when the fifteenth-generation seeds were grown at ambient CO_2_ in the reciprocal sowing experiment. In other words, *Arabidopsis* can positively respond to elevated CO_2_ within each generation, but elevated maternal CO_2_ had no significant effect on her offspring across 15 generations. Moreover, our study provides convincing evidence to confirm the assumption widely accepted in many previous studies that plant responses to elevated CO_2_ observed within a single generation are similar to those observed over many generations. Our results also suggest that future plants may not produce specific adaptation to increasing atmospheric CO_2_ concentrations due to the potential lack of genetic variation for CO_2_ responsiveness.

## Materials and Methods

### Experimental design

*Arabidopsis thaliana* plants of Wild-type Columbia (the Nottingham *Arabidopsis* Stock Centre, Nottingham University, Nottingham, UK) were continuously grown for fifteen generations, each generation lasting over 14 weeks. Plants were subjected to one of two treatments: (1) ambient CO_2_ (370 ppm) in each generation or (2) elevated CO_2_ (700 ppm) in each generation, following a well established protocol [27]. After each generation, we measured various reproductive, photosynthetic and cellular traits and compared those traits between the two CO_2_ treatments. In addition, the traits in each generation of the same treatment (ambient or elevated) were compared. Furthermore, we performed a reciprocal sowing experiment to test if *Arabidopsis* had evolved detectable adaptations to elevated CO_2_ at the end of fifteen generations.

### Selection and reciprocal sowing experiments

During the selection experiment, plants were grown for fifteen generations in two environment-controlled growth chambers. The seeds of *Arabidopsis thaliana* were first grown in the greenhouse, and seeds from the greenhouse-grown plants were used for the first generation. Generation m+1 was sown with seeds of plants from generation m (14≥m≥1) and 10% of the seeds from each individual plant were randomly selected and fully mixed for the next generation. To determine maternal responses at the end of fifteen generations in the selection experiment, we conducted a reciprocal sowing experiment. Seeds from the fifteenth generation at elevated CO_2_ were grown in both ambient and elevated CO_2_ growth chambers, as were seeds from the fifteenth generation at ambient CO_2_. For each generation, 35–45 plants were grown in each CO_2_ treatment. Plant growth and management followed a well established protocol [27].

Following previously described methods [24], [27], [34], [45], we used two chambers in the experiment: one chamber was controlled at 370±30 ppm and the other at 700±50 ppm. Throughout the experiment, other environmental factors including temperature, light, and relative humidity were identical in both growth chambers. The CO_2_ concentrations of the two chambers were swopped, and the pots were moved between chambers and randomly re-arranged weekly to negate any possible effects resulting from the chambers and pot position within the chambers and to minimize the potential for interactive effects between the chambers and developmental stages of plants.

### Determination of reproductive traits

For each generation, the number of days to reach first flowering was recorded for each plant. Plants were harvested after a 14-week growth period. The number of siliques per plant was determined by counting all intact siliques and central siliques that persisted after seed maturity [2]. The average length (up to 1 mm) of siliques was determined from 30 siliques randomly selected from each of ten plants in each treatment. The total number of seeds per plant was calculated as the total number of siliques per plant multiplied by the mean number of seeds per silique (determined from 30 randomly selected siliques per plant). After plant material was dried to a constant weight at 60°C, vegetative mass, reproductive mass and total mass were determined, respectively.

### Determination of stomatal, photosynthetic and cellular traits

When bolting had just commenced, i.e. at stage 5.10, fully expanded rosette leaves of plants in generation 1, 8, 15 and the reciprocal sowing experiment were respectively sampled for the analysis of stomatal density and leaf ultrastructure according to previous reports [27]. In addition, three fully expanded leaves from each of five plants were selected for the measurement of stomatal conductance, leaf transpiration rate as well as photosynthetic rate using an LI-6400 Portable Photosynthesis System (LI-COR Inc., Lincoln, Nebraska, USA). The measurements for ambient CO_2_-grown plants were carried out at 1500 µmol m^−2^ s^−1^ photosynthetically active radiation (PAR), 2.0–2.5 KPa vapour pressure deficit (VPD), 22–24°C and 380 ppm CO_2_, and for elevated CO_2_-grown plants at 1500 µmol m^−2^ s^−1^ PAR, 2.0–2.5 KPa VPD, 22–24°C and 700 ppm CO_2_.

### Statistics

The data are shown as mean±standard deviation. Data were subjected to one-way analysis of variance and *t*-test using software SPSS 10.0 (SPSS Inc., Chicago, IL, USA) and Excel 2003 (Microsoft Inc.).
